# To Punish or to Leave: Distinct Cognitive Processes Underlie Partner Control and Partner Choice Behaviors

**DOI:** 10.1371/journal.pone.0125193

**Published:** 2015-04-27

**Authors:** Justin W. Martin, Fiery Cushman

**Affiliations:** Department of Psychology, Harvard University, Cambridge, Massachusetts, United States of America; Wenzhou University, CHINA

## Abstract

When a cooperative partner defects, at least two types of response are available: Punishment, aimed at modifying behavior, and ostracism, aimed at avoiding further social interaction with the partner. These options, termed partner control and partner choice, have been distinguished at behavioral and evolutionary levels. However, little work has compared their cognitive bases. Do these disparate behaviors depend on common processes of moral evaluation? Specifically, we assess whether they show identical patterns of dependence on two key dimensions of moral evaluation: A person’s intentions, and the outcomes that they cause. We address this issue in a “trembling hand” economic game. In this game, an allocator divides a monetary stake between themselves and a responder based on a stochastic mechanism. This allows for dissociations between the allocator’s intent and the actual outcome. Responders were either given the opportunity to punish or reward the allocator (partner control) or to switch to a different partner for a subsequent round of play (partner choice). Our results suggest that partner control and partner choice behaviors are supported by distinct underlying cognitive processes: Partner control exhibits greater sensitivity to the outcomes a partner causes, while partner choice is influenced almost exclusively by a partner’s intentions. This cognitive dissociation can be understood in light of the unique adaptive functions of partner control and partner choice.

## Introduction

Cooperation with non-kin is a fundamental and ubiquitous feature of human society. Consequently, behavioral scientists have made an extraordinary effort to understand its foundations [1–7]. A dominant theme in this pursuit is the role of reciprocity in enforcing cooperative norms. In these “partner control models”, cooperation is maintained through the threat of punishment and the promise of reward [8]. Such threats are a reliable motivator of cooperation [9] and current research aims to describe the relationship between punishment and cooperation [4,9–14]. From this perspective, punishment and reward serve to encourage social partners to behave cooperatively in the future, thereby recouping their short-term costs.

Recently, however, an alternative path towards cooperation has attracted attention [15]. These “partner choice models” depend on the ability of organisms to choose whom to interact with [15–18]. It is assumed that, in many social interactions, interactants face a choice of “outside options”: Different (and potentially more cooperative) interaction partners. In any interaction in this population, if one partner defects, the other partner can simply switch to a new partner. This imposes a strong incentive for mutual cooperation because individuals who attempt to exploit their social partners become isolated and thereby fail to reap the benefits of mutually advantageous interactions. Partner choice models therefore tend to emphasize the role of reputation and fair exchange [15].

This distinction between partner choice and partner control provides insights into otherwise puzzling behavior. For instance, Krasnow and colleagues [13] found that when people experience defection from a social partner and choose to respond via punishment, they subsequently tend to re-initiate the same cooperative exchange—indeed, they are as trusting of a defector who they have punished as they are trusting of a person who cooperated all along. This behavior is explained by partner control models: Punishment is assumed to convert social partners from defectors to cooperators, and so the punisher seeks to re-establish cooperation. In contrast, when they choose not to respond with punishment, they tend to avoid future cooperative exchanges. This behavior is explained by partner choice models: Once a social partner is judged to be an inveterate defector, the best course of action is to cut one’s losses and simply avoid subsequent interaction with them. Thus, the distinction between partner control and partner choice provides an elegant explanation for the finding that people who punish a violation are more likely to subsequently trust than those who fail to punish the same violation.

Importantly, while previous work has distinguished between these models at the behavioral [13] and evolutionary levels [3,16–19], surprisingly little work has focused on the underlying cognitive processes. Although partner choice and partner control depend on different behaviors, it is possible that they are supported by a common set of psychological processes of moral evaluation. Alternatively, they may be supported by distinct and specialized processes.

Our approach to this question centers on a pair of cognitive processes that play a central role in moral evaluation: The assessment of an agent’s intent to cause harm, and of their causal role in harm coming about [20–27]. These factors can be dissociated in cases of accidental harm—instances where a person causes harm, but does not intend it. People tend to judge accidental harms more harshly than purely harmless actions, indicating sensitivity to outcomes. But, they also tend to judge accidental harms less harshly than malicious harms, indicating sensitivity to intent. This phenomenon has been noted by philosophers, who refer to it as moral luck [28,29]: When two people act with the same intentions but cause different outcomes, this element of luck influences our moral appraisals.

While it is widely agreed that assessments of intent and causal responsibility contribute to moral judgment, at least one study has found they do not matter equally for all types of judgments. Judgments of punishment (and blame) were shown to be more sensitive to outcome information than judgments of wrongness and permissibility [20]. In other words, moral luck is a special feature of punishment. This variability in outcome sensitivity provides a unique opportunity to probe the cognitive processes underlying partner control and partner choice. Punishment clearly belongs to the category of partner control strategies. It is less obvious whether appraisals of “moral wrongness” and “moral permissibility” derive from strategies of partner choice, however. Our aim is to design an experimental context that unambiguously selects for partner control versus partner choice strategies and to investigate the reliance on outcome versus intent information for each.

To accomplish this, we employ an economic game adapted from Cushman and colleagues [26]. Paired participants are allocated a stake of money and one of them, dubbed the Roller, allocates this stake by rolling one of two dice. One die is likely to lead to a selfish split of the money, favoring the Roller, while the other is likely to lead to an even split. Importantly, either die can produce either outcome—the Roller can roll the selfish die but still bring about a fair split—dissociating the Roller’s intent and the obtained outcome. The other participant in each pair, called the Responder, responds to the Roller’s die choice and the outcome of the roll in one of two ways. In the Partner Control condition, Responders punish or reward the Roller by attempting to take or grant a sum of money. In the Partner Choice condition, Responders decide whether, on a subsequent round, they want to play again with the same Roller or switch to a new Roller. Thus, we vary the Roller’s intent and the obtained outcome, as well as whether responses constitute partner control or partner choice. Consistent with prior research [20,26], we hypothesize that punishment behavior will show substantial sensitivity to both outcome and intent information. In contrast, we hypothesize that partner choice will rely exclusively on information about intent. This is predicted because, presumably, a person’s intentions are a more reliable indicator of their future behavior than the accidental outcomes that they happen to bring about.

Of course, our hypothesized result immediately raises the question of *why* punishment is especially sensitive to moral luck. Phrased more generally: Why might partner control be particularly sensitive to the outcome caused, while partner choice shows a relatively larger sensitivity to an agent’s intentions?

The dominant approach to moral luck in the psychological literature characterizes it as a bias, either stemming from the salience or emotional impact of the bad outcome itself [30–32] or else from the tendency to assume that a person’s intent is malicious when they cause a bad outcome [33–38]. However, these approaches do not provide a natural explanation for the unique relationship between punishment and moral luck. If moral luck is a psychological bias, we would expect it to apply equally to all judgment types and contexts: wrongness and permissibility as well as punishment, and partner choice as well as partner control.

In behavioral economics, an impact of outcomes is often observed due to the motive for distributional equality—that is, a tendency to prefer outcomes that are equitable and fair [39,40]. Thus, outcome-based punishment might stem from a desire to equate payoffs between partners, by reducing the higher payoff. Cushman and colleagues [26] found, however, that distributional concerns cannot fully explain the impact of outcomes on punishment decisions. Specifically, they found that punishment was sensitive to outcomes, even when the cause of an outcome had no control over it (e.g. a partner rolled a die that caused a random outcome), consistent with a distributional explanation. However, when the partner had some measure of control over the final outcome—they chose between 3 die, each varying in the likelihood of a good outcome—more punishment was assigned, and this effect was independent of any impact that intentions had. Thus, concerns about fairness or equity cannot fully account for the influence of outcomes.

Here, we posit an explanation for the punishment of accidents that rests on the adaptive function of punishment: Pedagogy [41]. Ultimately, punishment will be favored when it successfully changes others’ behavior in a way that improves one’s own fitness [4,9,42]. This can explain why punishment is more sensitive to outcomes than other judgments: Even in the absence of a bad intent (e.g. in the case of accidental harm), punishment of a bad outcome sends a signal to the one punished that they should change their behavior to avoid that outcome in the future. In essence, an accident is a teachable moment [43]: Although a person whose behavior is costly to you may not be intending harm, sanctioning them can still send a signal to behave better in the future.

Importantly, our argument is not that the motivation for pedagogical punishment is deliberative or rational. Indeed, work on the psychological motivations for punishment find that they are largely retributive [31,32,44]. Rather, we suggest that retributive impulses may be shaped, to some degree, towards pedagogy [43]. In this sense, while outcome-based punishment may on the whole have the effect of teaching partners to behave more beneficially, the retributive impulses that guide pedagogical punishment may misfire in certain situations. Critically, such errors will be specific to punishment and should not be present for other behavioral responses, including partner choice, that do not serve a pedagogical function.

This distinction between the ultimate and proximate basis of punishment is especially important because the punishment of accidents in our experiment seems ill-suited to teaching partners. A Roller who chooses the fair die that nonetheless happens to come up selfishly has taken the most prosocial action possible. Thus, there is no need for teaching. (Indeed, punishment might actually lead toward less subsequent prosocial behavior). Consistent with prior research [26] we argue that the punishment of accidental outcomes has value at the ultimate level, but that the proximate mechanism is overgeneralized in the specific context of our experiment.

In sum, we set out to test the degree to which partner control and partner choice behaviors share an underlying cognitive basis. Doing so requires an experimental paradigm with three features: (1) An allocator can choose between different allocation amounts, of varying benefit to herself and a responder, (2) that the allocator has only imperfect control over the eventual outcome, so that her intent can be divorced from the obtained outcome (e.g. she can try to be prosocial, but actually end up causing a selfish outcome), and (3) that responses available to responders conform to either partner control or partner choice models.

## Materials and Methods

### Participants

All participants were recruited through an online study pool and participated for partial course credit, with the possibility to earn an additional monetary bonus. Participants (N = 117, 27.8% male, mean age = 19.2 years, range 18–23 years) provided written informed consent and all methods were approved by the Brown University Institutional Review Board.

### Design

The design was closely modeled on prior work [26]. Participants played an economic game in groups of at least 4 but no more than 12 people (median group size = 7), with all participants seated at the same table in the same room. Participants were assigned to one of two rolls in the game, with roughly half playing as a Roller and the other half playing as a Responder. Participants were anonymously matched with each other in pairs of one Roller and one Responder. In the event of an uneven number of participants, the odd participant was assigned to be a Responder and one of the Rollers unknowingly played two games (i.e. the Roller’s choice and die outcome was made known to two Responders, who were also unaware of this, but the Roller was only given feedback about one of the games). Thus, participants knew they were playing against someone else in the room, but not who that person was.

In each pair, the Roller’s job was to allocate $4 per round between themselves and the Responder. To do this, they selected and rolled one of two 6-sided dice: A selfish die (S) or a fair die (F) (during the experiment the dice were referred to by the letters A and B). Die S had a 2/3 chance (numbers 1–4) of giving $4 to the Roller and $0 to the Responder (a selfish outcome), and a 1/3 chance (numbers 5–6) of giving $2 to each player (a fair outcome). Die F had the opposite probabilities: 2/3 chance of an even $2 split (numbers 1–4), and a 1/3 chance of giving all $4 to the Roller (numbers 5–6). Both the Responder and the Roller were instructed as to these payoff contingencies and were given an instruction sheet with this information that was continuously available. Thus, the Roller’s intent could be perfectly inferred by their choice of die: A selfish Roller would choose die S, which was more likely to give the money to themselves, while a prosocial Roller would choose die F, which was more likely to give a fair split. Importantly though, the Roller only had imperfect control over the eventual outcome: Despite choosing the fair die, the roll was likely to yield a selfish split 1/3 of the time.

The Responder indicated how they wanted to respond to every possible combination of the Roller’s choice and the roll of the die: A fair or selfish choice, crossed with a fair or selfish outcome. The response options differed by condition. In the Partner Control condition, Responders were able to subtract or add up to $2 to the Roller’s final outcome. This punishment or reward was never referred to as such, but rather as “additions and subtractions”, so as to minimize demand characteristics. Adding or subtracting was costless for the Responder. Additionally, additions and subtractions were imposed with 1/12 probability (11/12 of the time, no addition or subtraction actually occurred). This was done so that Rollers’ decisions would stem from genuine prosocial or antisocial motivations, rather than calculated attempts to maximize reciprocated payoffs. In the Partner Choice condition, Responders were able to choose whether they would like to play another round with the same Roller, or whether they would like to switch to a new Roller from among the other players. This decision was deterministic: A choice to switch Rollers meant that the participant would definitely play with a new Roller on the next round.

Because we wanted Responders’ decisions to stay or switch in the Partner Choice condition to be real, the Partner Choice condition required the use of 2 rounds, whereas only one was needed in the Partner Control condition. In the Partner Choice condition, Rollers and Responders were instructed that two rounds of the game would be played, with the second played in the same manner as the first (e.g. Rollers would choose which die to roll, and Responders would choose to stay or switch, though this latter choice was only hypothetical in the second round). In a pilot version of this task we explored a version of the Partner Control condition that also included 2 rounds of play. In this case, Responders almost exclusively adopted an indifferent strategy, failing to reward or punish. Debriefing indicated that participants were unwilling to punish a partner who they would subsequently interact with, in anticipation of retaliatory defection.

So as to obtain the largest amount of useable data, the strategy method was used: Participants indicated for both possible die choices, die S and die F, and the possible outcomes, a fair or selfish split of the money, how they would like to respond. This allowed us to obtain four responses per Responder.

### Procedure

Participants were provided with written and verbal descriptions of the game and given ample time to ask questions. Both Rollers and Responders used worksheets to indicate their choices. Barriers were erected between all players so that no one could see the choices selected by other players. Rollers indicated their die choice and were then given and rolled this die, the outcome of which was recorded by the experimenter. Once all responses were collected, the experimenter used the worksheets to communicate the outcome of the die roll to the Responders and then communicated the Responders’ choices (to either add/subtract money from the Roller and whether this was successful, or to stay with the current Roller or switch to a new one) to the Roller. In resolving impossible decisions to switch in the Partner Choice condition (e.g. only one Responder decides to switch), participants were informed that switches occurred, though they continued to be paired with the same partner on the 2^nd^ round (a decision that was impossible to implement occurred in only 4 out of 31 interactions in the Partner Choice condition). Importantly, as all worksheets were kept hidden from other participants and the mapping between Roller and Responder was only known to the experimenter, Responders had no way of knowing who they were actually playing with. Rollers completed an additional step, where they were asked to play the game as a hypothetical Responder and given the same worksheet that Responders filled out. Participants were then told their final outcome, paid and debriefed.

## Results

As responses in the Partner Control and Partner Choice condition are qualitatively different (a dollar amount in the Partner Control condition and a categorical choice to switch or stay in the Partner Choice condition), we first assess the overall sensitivity to intentions and outcomes within each condition, and then analyze the proportion of participants exhibiting sensitivity to these factors across conditions. Specifically, within each condition we determined the proportion of participants conditioning their response on the Roller’s die choice and the proportion conditioning their response on the outcome of the die. We also determined the proportion of participants exhibiting sensitivity to neither factor (responding uniformly across all four conditions). We then compare the proportion of participants showing sensitivity to these factors between the two conditions.

### Partner Control condition

**Fig 1:**
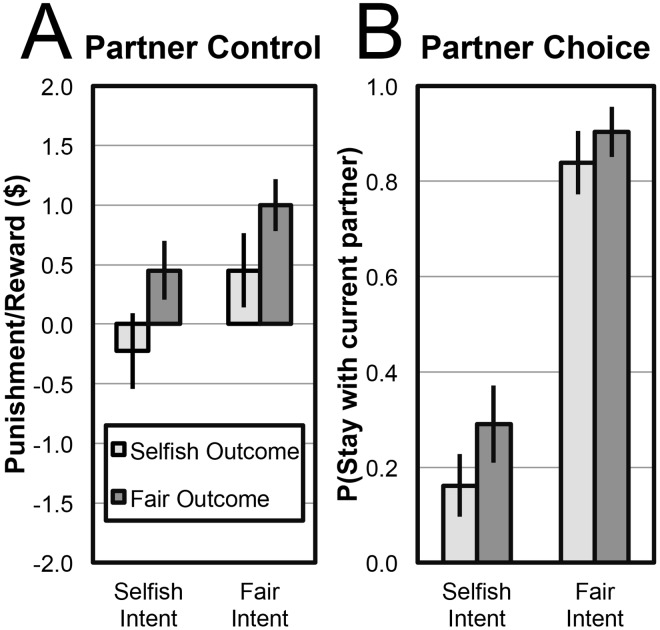
Mean Responder decisions to each combination of intent (choice of die) and outcome (allocation amount) in the (A) Partner Control and (B) Partner Choice conditions. Responders’ decisions in each condition as a function of the Rollers’ intent and result of die roll. (A) Responders’ allocations in the Partner Control condition. Positive values indicate reward, negative values indicate punishment. Error bars are SEM. (B) Probability of a Responder in the Partner Choice condition indicating a desire to switch to a new Roller. Lower values indicate a desire to switch. Error bars are SE of proportion. Legend applies to both graphs.

Participants in the Partner Control condition demonstrated strong sensitivity to both intention (die choice) and outcome. Fourteen of the 31 Responders demonstrated sensitivity to intentions: Rewarding more or punishing less when the Roller choose die F. The same proportion of participants (14/31) conditioned their response on outcomes, rewarding more or punishing less for equal splits of the $4. Thus, these factors exert equal influence on partner control behavior. Finally, a subset of participants (12/31) did not exhibit sensitivity to either factor, responding in a uniform fashion across all four conditions. While one always punished the full $2 and two left the Roller’s payoff unchanged, the majority (9) always rewarded the full $2. The average amount of punishment and reward conditional on the Roller’s intent and the obtained outcome is plotted in Fig 1A.

We also modeled participants’ behavior using mixed-effects maximum likelihood regression, implemented in Stata 13.1 [45]. We regressed Responders’ responses on mean-centered fixed-effects predictors for intent, outcome and their interaction, as well as a random-effects predictor coding for subject. We find large fixed effects for intent (**β** = 0.61, SE = 0.19, p<0.005) and outcome (**β** = 0.61, SE = 0.17, p<0.001). Once these main effects are accounted for, the interaction term provides no additional explanatory power (**β** = -0.13, SE = 0.35, p = 0.71). This analysis confirms that both intentions and outcomes exert a large and roughly equivalent influence on participants’ punishment and reward determinations.

Looking at the behavior of the Rollers, we find that the majority (23/28) chose to roll the selfish die, with the remainder rolling the fair die. This suggests that Rollers correctly understood the economic payoffs of the game, were cognizant of the fact that punishment and reward were only infrequently applied and were not strongly influenced by reputational concerns. This also suggests that Rollers in this condition were not intrinsically motivated to be pro-social (see Discussion for elaboration of this point).

### Partner Choice condition

Participants in the Partner Choice condition exhibited a starkly different pattern of results, switching to a new partner largely exclusively on the basis of the current Roller’s intentions. Of the 31 Responders, 24 exhibited sensitivity to the Roller’s intentions, switching to a new partner when die S was chosen and staying with the current partner when die F was chosen. Only eight participants demonstrated sensitivity to the outcome of the die roll, switching for unfair splits and staying for fair splits. Finally, three participants showed a uniform pattern of results, either always staying with the current Roller (2) or always switching (1). The overall probability of staying with the same Roller or switching to a new one based on the chosen die and the obtained outcome is plotted in Fig 1B.

We again turn to regression analyses to provide a fuller picture of how participants’ responses are influenced by intentions and outcomes within this condition. Here, as responses are categorical in nature, we used mixed-effects logistic regression. Responses were regressed on mean-centered fixed-effects predictors for intent, outcome and their interaction, as well as a random-effects predictor coding for subject. First, we find a significant effect of intent (**β** = -3.47, SE = 0.45, p < 0.001). The effect of outcomes on decisions to switch to a new partner was marginal (**β** = -0.67, SE = 0.37, p = 0.072). No significant effect was found for the interaction term (**β** = -0.03, SE = 0.74, p = 0.97). Here, confirming the proportions above, we find a large effect of intentions on decisions to switch to a new partner, and only a marginal impact of outcomes.

Roughly half of the Rollers in the Partner Choice condition (13/27) choose the selfish die, with the other half choosing the fair die, a pattern significantly different from the behavior evident in the Partner Control condition (Fisher’s Exact Test p < 0.05, N = 55). Why might Rollers in the Partner Choice condition have decided to be more pro-social? One potential explanation is that the possibility of a Responder switching to a new Roller is more aversive than the possibility of an unlikely $2 punishment.

### Comparative analyses

In order to analyze the relative sensitivity to the Roller’s intent and the obtained outcome between the Partner Control and Partner Choice conditions, the proportion of Responders exhibiting sensitivity to each factor was compared between conditions. This analysis is complicated, however, by a third group of participants: Responders who showed sensitivity to neither intentions nor outcomes. The proportion of participants in this “insensitive” group differs significantly between the Partner Control and Partner Choice conditions (Partner Control: 38.7%; Partner Choice: 9.7%; Fisher’s Exact Test p < 0.05, N = 62). Why were participants approximately four times as likely to exhibit a uniform response in the Partner Control condition? In this condition, 9 out of the 12 insensitive participants gave the highest possible amount of money ($2) regardless of the Roller’s intent or the outcome of the die. These prosocial intentions are well-captured by a representative quote from a participant asked to articulate her thought process while deciding how much to give to or take from the Roller’s payoff: “It didn’t affect me, so why not be generous and try to help them out.” Six of these 9 participants (67%) made similar comments, demonstrating a focus on benefiting their partner above all else.

Because participants in the Partner Control condition were much more likely to exhibit insensitivity to both outcome and intent, this lowers the proportion of participants exhibiting sensitivity to either of the two factors. We can correct for this unwanted influence by simply excluding those participants exhibiting insensitivity. In essence, we would then be asking, “among those participants who exhibit sensitivity to *either* outcome or intent in each condition, which form of sensitivity is more likely?” In order to take a maximally conservative approach, we analyze our data both ways: First excluding participants who exhibit insensitivity, and then including them.

First, excluding insensitive participants, we find no difference in the proportion of participants exhibiting sensitivity to intentions between the Partner Control and Partner Choice conditions (Partner Control: 73.7%; Partner Choice: 85.7%; Fisher’s Exact Test p = 0.45, N = 47). Looking at outcomes, we find a different pattern: More participants in the Partner Control condition are sensitive to outcomes (Partner Control: 73.7%; Partner Choice: 28.6%; Fisher’s Exact Test p < 0.005, N = 47). In sum, whether responding is limited to partner control or partner choice methods has no impact on how strong a role intentions play, but does change the weight that is given to outcomes.

Including participants who were insensitive to both intentions and outcomes reveals a different pattern of results. We now find a difference in relative sensitivity to intentions between the conditions (Partner Control: 45.2%; Partner Choice: 77.4%; Fisher’s Exact Test p < 0.05, N = 62). For outcomes, there is no significant difference (Partner Control: 45.2%; Partner Choice: 25.8%; Fisher’s Exact Test p = 0.18, N = 62).

These analyses converge on the conclusion that participants show differential sensitivity to outcome versus intent across the Partner Choice and Partner Control conditions. Although the patterns of significance and non-significance differ between the two analyses, we see compatible trends in each case: Greater reliance on outcomes, and lesser reliance on intent, in the Partner Control condition compared with the Partner Choice condition.

To confirm these trends, we repeated our analysis while including responses from Rollers who indicated their hypothetical patterns of response for the Responder role. One Roller failed to complete this sheet, leaving data from 54 Rollers and 62 Responders (116 participants total). First, and critically, we found no significant differences between Rollers’ hypothetical responses and Responders’ actual behavior in intent sensitivity (Partner Control: Responders—45.2%, Rollers—32.1%, Fisher’s Exact Test p = 0.42, N = 59; Partner Choice: Responders—77.4%, Rollers—80.8%, Fisher’s Exact Test p = 1, N = 57), outcome sensitivity (Partner Control: Responders—45.2%, Rollers—50.0%, Fisher’s Exact Test p = 0.80, N = 59; Partner Choice: Responders—25.8%, Rollers—30.8%, Fisher’s Exact Test p = 0.77, N = 57) or indifference (Partner Control: Responders—38.7%, Rollers—39.2%, Fisher’s Exact Test p = 1, N = 59; Partner Choice: Responders—9.7%, Rollers = 0%, Fisher’s Exact Test p = 0.24, N = 57).

Based on this similarity, we combined Rollers’ and Responders’ data and repeated the analyses described above. We again find differences in the proportion of insensitive participants between conditions (Partner Control: 39.0%, Partner Choice: 5.4%; Fisher’s Exact Test p < 0.001, N = 116). However, excluding these participants no longer changes the overall pattern of results. In either case, we find a greater proportion of participants demonstrating sensitivity to intent in the Partner Choice condition (Excluding insensitive: Partner Control—63.9%, Partner Choice—83.3%, Fisher’s Exact Test p < 0.05, N = 90; Including insensitive: Partner Control—39.0%, Partner Choice—78.9%, Fisher’s Exact Test p < 0.001, N = 116). For outcomes, we find the opposite pattern: Regardless of whether insensitive participants are excluded or not, a greater proportion of participants are sensitive to outcomes in the Partner Control condition (Excluding insensitive: Partner Control—77.8%, Partner Choice, 29.6%, Fisher’s Exact Test p < 0.001, N = 90; Including insensitive: Partner Control—47.5%, Partner Choice—28.1%, Fisher’s Exact Test p < 0.05, N = 116).

Thus, including data from Rollers’ (which was no different from the behavior of Responders) boosts power sufficiently to detect an effect of response type on both outcome sensitivity and intent sensitivity. In line with our hypotheses, partner control exhibits greater sensitivity to outcomes, while partner choice is influenced more strongly by intentions.

## Discussion

This study investigates the cognitive underpinnings of two behavioral strategies that support human cooperation: Partner choice and partner control. We find clear evidence for systematic differences in the degree of reliance on information about accidental outcomes. Partner choice decisions depended overwhelmingly on information about a partner’s intentions, and not on the obtained outcome. In contrast, punishment decisions depend jointly on both intention and outcome.

These findings contribute in several ways to our understanding of the functional and mechanistic basis of social behavior. At the most basic level, they provide an important complement to past studies indicating that different types of moral judgment (deserved punishment, permissibility, character, etc.) differ systematically in their reliance on information about outcome versus intent [20]. Past research relied on self-report judgments of hypothetical dilemmas, whereas the present study evaluates participants’ actual behavior in an incentivized context. In the present study we assessed behaviors (punishment versus social exclusion) rather than categories of moral judgment. We find that social exclusion—a variety of partner choice behavior—appears to share a cognitive basis with wrongness, permissibility and character, each of which depends nearly exclusively on intentions rather than outcomes. Meanwhile, punishment shows a strong reliance on outcome alongside intent. Additionally, the present study directly assesses behavior in paradigm cases of partner choice versus partner control, allowing us to more directly link evidence about the mechanisms of moral judgment with theories of their adaptive rationale.

More broadly, our results have implications for understanding how and why humans cooperate. It is disputed whether both partner choice and partner control mechanisms account for the extraordinary breadth of cooperative activities in humans, or whether one or the other mechanism is sufficient [15]. Insofar as we have identified distinct cognitive architectures adapted for each circumstance, this suggests that both mechanisms likely played an important role in human social life. Moreover, we can use the distinct cognitive fingerprints of partner choice and partner control mechanisms to determine which is operative in different experimental or everyday contexts. For instance, a recent study found that reciprocity decisions were overwhelmingly intent-based in a stochastic iterated prisoner’s dilemma [46]. In principle the iterated Prisoner’s Dilemma might be conceptualized as instantiating partner control (e.g. cooperation conveys a benefit while defection imposes a cost), or instead as partner choice (e.g. defecting early in an interaction may indicate a lack of trust, effectively withdrawing from future interactions). Our findings suggest that the observed intent-based pattern of reciprocity is likely the consequence of participants construing this game in terms of partner choice, rather than partner control.

Finally, our findings help to constrain the available explanations for “moral luck”, or the tendency to make outcome-based moral judgment. Existing psychological theories of moral luck have tended to argue that it reflects an error or bias in judgment [30–32,36–38,47,48]. On this account, the mere presence of a negative outcome biases subsequent responding in one of two potential ways. On an “outcome bias” account, perception and evaluation of a negative outcome generates negative affect, and this heightened emotionality then biases moral judgment as a side effect. On a “hindsight bias” account, the presence of a negative outcome causes a perceiver to re-evaluate the agent’s mental state (e.g. If he caused the car accident, he must have been driving recklessly), and this harsher evaluation of his intent leads to harsher moral judgment. However, it is hard to explain why a pervasive error would occur for some judgments (punishment and reward) but not for others (choosing to continue or end a relationship).

Rather, our data favors an adaptive explanation for moral luck grounded in the unique function of punishment: to modify social partners’ future behavior by exploiting their capacity for learning. Punishing based on outcomes—even when unintended—may be adaptive because of its communicative value. A social partner may be unaware that they are harming you, and in this sense the harm is unintended; nevertheless, punishing that behavior can send an important signal to the social partner to change their behavior. While partner control models are predicated on teaching one’s partner and changing their behavior, partner choice models need not be sensitive to signaling concerns. Rather, partner choice models focus principally on whether that agent will continue to be cooperative in the future, and the best index of this is their demonstrated intentions.

Of course, the logic of partner control does not apply perfectly to the current paradigm because it was a one-shot and anonymous interaction: There was no need for participants to “teach” their partner, both because they would not play with them again, and because the partner actually chose the best possible option given the experimental constraints. In fact care was taken to minimize any reputational benefits from responses. Rather, we propose that the mechanistic basis for punishment does not perfectly recapitulate its ultimate, adaptive rationale. Prior work shows that punitive motivations are largely retributive, rather than deterrent, in nature [31,32,44]. Nevertheless, mechanisms of retributive punishment may still serve the ultimate adaptive purpose of deterrence.

This divergence between proximate mechanism and ultimate function may be favored for at least two reasons. First, reasoning about the deterrent value of punishment requires time and effort that can be saved by relying on a heuristic. Similar arguments have been advanced for the proximate versus ultimate basis of cooperation [49,50]. Second, reasoning about the deterrent value of punishment exposes the reasoner to exploitation and deception. Specifically, a social partner can respond to punishment by obstinately refusing to learn. A reasoning punisher would recognize the futility of engaging in further costly punishment and succumb to this manipulation. In contrast, a blind heuristic for retribution based on outcomes cannot be undermined in this manner. Consistent with this analysis, emotions like revenge have been modeled as strategically rational commitments to tactically irrational behavior [51]. Of course, relying on a blind heuristic carries a key disadvantage: a lack of flexibility that can lead to “errors” in the application of punishment, such as in the specific context of our experiment.

Two features of our data were not predicted *a priori* and deserve further attention. First, we found a disproportionate number of participants employing an indifferent strategy in the Partner Control condition (39%) compared to the Partner Choice condition (10%). This complicates our analysis of the differential reliance on outcome versus intent, and it stands out as an important feature of the data to explain. We conjecture that participants in the Partner Control condition, where giving or taking money was the option available to them, noticed an opportunity to give money to someone in their community at no cost to themselves. This is in line with recent work demonstrating especially high allocations in the Dictator Game in the US [52]. Consistent with this explanation, 75% of participants who adopted a uniform response in the Partner Control condition chose to give money to their partner uniformly across conditions, compared with 17% who gave no money and 8% who deducted money. Participants in the Partner Choice condition had no such opportunity for unilateral charity because neither staying nor switching afforded any advantage to their partner.

Second, Rollers in the Partner Choice condition were much more likely to choose the fair die than Rollers in the Partner Control condition (52% versus 18%, respectively). In other words, being assigned to the Partner Choice condition prompted *Rollers* to behave more charitably. This finding is especially unexpected because in the Partner Control condition Rollers have some economic incentive towards fair behavior (which can be rewarded), whereas in the Partner Choice condition there is no such incentive. However, it is important to recall that we intentionally designed our experiment such that Rollers’ decisions were very weakly incentivized by Responder behavior in the Partner Control condition: punishment and reward was only effective 1/12 of the time, and the maximum amount of punishment or reward was half the total amount possibly earned.

One interpretation of this finding is that people find the possibility of rejection by a social partner more motivating than the possibility of punishment, despite the fact that the former carries no financial costs in the context of our experiment. In more representative ecological contexts this may reflect a very rational concern: Punishment imposes a “one time” cost on its target, whereas ostracism imposes a cost that may continue indefinitely and grow to a much larger magnitude. This highlights the potential use of the *threat* of ostracism (an act of partner control) as a means to modify partners’ behavior (an effect of partner control), potentially blurring the lines between the two adaptive rationales for prosocial behavior that we consider here.

A second possible explanation for differing levels of fairness across the Partner Control and Partner Choice conditions is motivational crowding. Much past research shows that small extrinsic incentives for social behavior can undermine, or “crowd out”, larger intrinsic incentives, thereby reducing the performance of the behavior overall (for review, see [47]; [53–55]). In the Partner Choice condition, Roller behavior is dependent upon intrinsic motivations for prosociality exclusively. But, in the Partner Control condition, the possibility of a punishment or reward based on their behavior introduces a weak extrinsic motivation, potentially crowding out intrinsic motives. We cannot distinguish between these accounts based on the current data.

## Conclusion

Understanding how and why humans cooperate is a major challenge for the behavioral sciences. Partner control and partner choice models have been investigated from behavioral and evolutionary standpoints, but relatively little work has explored their cognitive underpinnings. The present work takes up this challenge, finding that partner control and partner choice models of behavior are supported by at least partially distinct cognitive mechanisms. Specifically, the former is more sensitive to moral luck and the influence of outcomes than the latter. We have argued that the unique role of moral luck in partner control is best explained in terms of its unique pedagogical function, although certainly the present study is not decisive on this point. Rather, our findings invite further investigation of how humans flexibly employ partner control and partner choice behaviors to maintain cooperative equilibria.

## Supporting Information

S1 File Partner Control condition materials. Materials used in the Partner Control condition.
(DOC)

S2 File Partner Choice condition materials. Materials used in the Partner Choice condition.
(DOC)
