# Supplementary material for: To Punish or to Leave: Distinct Cognitive Processes Underlie Partner Control and Partner Choice Behaviors
Source: PLoS One. 2015 Apr 27;10(4):e0125193. doi: 10.1371/journal.pone.0125193 (PMC4411127; doi:10.1371/journal.pone.0125193)
Supplement: S1 File — Materials used in the Partner Control condition. (DOC) [file pone.0125193.s001.doc]

**Experiment Script**

Subjects are welcomed and guided to opposite sides of the room in alternating order.

Experiment welcomes subjects, says:

- You will be handed instructions, they are complicated, please read them carefully.
- Experimenter will go over things verbally, please wait until after that to ask questions.
- Please try to keep a “poker face” during this experiment, the only interactions between participants should be on paper handed back and forth by experimenters.

All subjects receive instructions, read them.

Experimenter reviews instructions, saying:

- In this game you will interact with one other person in this room now, but that interaction will be completely anonymous.
- People are paired by roller/responder – identify which side of the room is which.
- Roller must allocate $4 between self and responder
- Choice of two die, explain how the die work
- Responder can attempt to add/subtract money from roller (or not – can leave unchanged)
- *Totally up to responder to decide. Decide for yourself.*
- Attempt only works 1/12 of time.
- Responder must make decision for all possible outcomes in advance
- We collect forms, work out what happens, and report back to both players

All subjects receive response sheet for round 1 and fill it out.

Experimenters collect sheets, compute outcomes, and return feedback sheets.

Everyone is paid in an envelope and leaves.

Thank you for participating in this experiment.

Please read the following instructions very carefully. If anything is unclear, please ask the experimenter for clarification.

In this experiment, pairs of people interact in a simple game. During this interaction (or “round”), one subject divides $4 between themselves and the other subject. This person is called the “roller” (you’ll see why, soon). The other subject then responds to that division. This person is called the “responder”.

Today, you are going to play the role of a “responder”. We will randomly pair you with one of the rollers who is in the room right now. However, you do not know which person is your roller, and the roller does not know which responder you are. We will keep your identity, and the roller’s identity, completely anonymous.

At the beginning of the game, there is $4 to be divided between you and the Roller. The Roller will determine how to make this division. In order to do so, the roller can choose which of two die to roll: A or B. The roller can choose either one. Here’s how the die work:

Die A: If the roller rolls a 1, 2, 3 or 4, then the Roller gets all $4.

If the roller rolls a 5 or 6, then the Roller gets $2 and you get $2.

Die B: If the roller rolls a 1, 2, 3 or 4, then the Roller gets $2 and you get $2.

If the roller rolls a 5 or 6, then the Roller gets all $4.

You have the chance to respond. Specifically, you can attempt to add to or subtract from the Roller’s payoff: up to $2 added, or up to $2 subtracted, or anything in between. It will not cost you anything to add or subtract money from the Roller. Any money that is subtracted from the Roller’s payoff will be returned to the experimenter. So, these additions and subtractions can affect the Roller, but not you.

However, there is only a 1/12 chance that your attempt to add or subtract money will succeed. Chances are 11/12 that your attempt will fail, and the Roller's payoff will not be affected at all. Therefore, the probability that money will be added or subtracted from the Roller's payoff is very low. The Roller knows that the probability of successful addition or subtraction is only 1/12. The Roller will be informed of your attempt to add or subtract money, even if your attempt ends up being unsuccessful.

The experimenter will use a randomized procedure to determine whether your attempt will succeed or not. Whether your attempt succeeds has nothing to do with the behavior or choices of either you or the Roller.

Please turn the page.

Responder Subject ___

**For you to fill out:**

You must first decide how you will respond to each possible outcome. You can add or subtract up to $2 from the Roller’s payoff, or anything in between. Circle "+" or "-" to indicate whether you are adding or subtracting, and then fill in the amount that you want added or subtracted.

Roller’s The die Roller Addition or

choice: comes up: gets: You get: subtraction:

Die A 1, 2, 3 or 4 $4 $0 $ + / - ____

Die A 5 or 6 $2 $2 $ + / - ____

Die B 1, 2, 3 or 4 $2 $2 $ + / - ____

Die B 5 or 6 $4 $0 $ + / - ____

**For the experimenter to fill out:**

The Roller chose die: ______

The die came up: ______

Your attempted response was: ______

This response did / did not affect the roller’s payoff.

Subject ___

Some Final Questions

1. Which die would you choose if you were the Roller? Why would you make that choice?

2. Which die did the Roller choose? Why do you think they made that choice?

3. What were the factors you considered when deciding whether to add or subtract money from the Roller?

4. We would like to know whether you found any of the decisions about adding or subtracting money from the Roller's payoff to be particularly difficult. Please put a check mark by any decision(s) you found particularly difficult:

Roller rolls: The die comes up: Roller gets: You get: Difficulty:

Die A 1, 2, 3 or 4 $4 $0 ______

Die A 5 or 6 $2 $2 ______

Die B 1, 2, 3 or 4 $2 $2 ______

Die B 5 or 6 $4 $0 ______

5. What is your gender? Please circle one: Female Male

6. What is your age in years? _____

7. What is your occupation? If you are a student, what is your primary field of study?

Thank you for participating in this experiment.

Please read the following instructions very carefully. If anything is unclear, please ask the experimenter for clarification.

In this experiment, pairs of people interact in a simple game. During this interaction (or “round”), one subject divides $4 between themselves and the other subject. This person is called the “roller” (you’ll see why, soon). The other subject then responds to that division. This person is called the “responder”.

Today, you are going to play the role of a “roller”. We will randomly pair you with one of the responders who is in the room right now. However, you do not know which person is your responder, and the responder does not know which roller you are. We will keep your identity, and the responder’s identity, completely anonymous.

At the beginning of the game, there is $4 to be divided between you and the responder. You will determine how to make this division. In order to do so, you can choose which of two die to roll: A or B. You can choose either one. Here’s how the die work:

Die A: If you roll a 1, 2, 3 or 4, then you get all $4.

If you roll a 5 or 6, then you get $2 and the responder gets $2.

Die B: If you roll a 1, 2, 3 or 4, then you get $2 and the responder gets $2.

If you roll a 5 or 6, then you get all $4.

The responder will have the chance to respond. Specifically, the responder can attempt to add to or subtract from your payoff: up to $2 added, or up to $2 subtracted, or anything in between. It will not cost the responder anything to add or subtract money from you. Any money that is subtracted from your payoff will be returned to the experimenter. So, these additions and subtractions can you, but not the responder.

However, there is only a 1/12 chance that the responder’s attempt to add or subtract money will succeed. Chances are 11/12 that the responder’s attempt will fail, and your payoff will not be affected at all. Therefore, the probability that money will be added or subtracted from your payoff is very low. You will be informed of the responder’s attempt to add or subtract money, even if that attempt ends up being unsuccessful.

The experimenter will use a randomized procedure to determine whether the responder’s attempt will succeed or not. Whether the responder’s attempt succeeds has nothing to do with the behavior or choices of either you or the responder.

Please turn the page.

Roller Subject ___

**For you to fill out:**

Please indicate which die you would like to roll. Circle one: **Die A Die B**

After you roll the die, please write down the number that came up here: ______

**For the experimenter to fill out:**

In Round 1, you chose die: ______

The die came up: ______

The responder’s attempted response was: ______

This response did / did not affect your payoff.

Subject ___

Some Final Questions

1. Which die did you choose? Why did you make that choice?

2. How did the other player respond to your choices? What factors do you think the other player was considering, and how did they make their decisions?

3. What is your gender? Please circle one: Female Male

4. What is your age in years? _____

5. What is your occupation? If you are a student, what is your primary field of study?

Roller as Responder Subject ___

**For you to fill out:**

You must first decide how you will respond to each possible outcome. You can add or subtract up to $2 from the Roller’s payoff, or anything in between. Circle "+" or "-" to indicate whether you are adding or subtracting, and then fill in the amount that you want added or subtracted.

Roller’s The die Roller Addition or

choice: comes up: gets: You get: subtraction:

Die A 1, 2, 3 or 4 $4 $0 $ + / - ____

Die A 5 or 6 $2 $2 $ + / - ____

Die B 1, 2, 3 or 4 $2 $2 $ + / - ____

Die B 5 or 6 $4 $0 $ + / - ____

Subject ___

Some Final Questions

1. What were the factors you considered when deciding whether to add or subtract money from the Roller?

2. We would like to know whether you found any of the decisions about adding or subtracting money from the Roller's payoff to be particularly difficult. Please put a check mark by any decision(s) you found particularly difficult:

Roller rolls: The die comes up: Roller gets: You get: Difficulty:

Die A 1, 2, 3 or 4 $4 $0 ______

Die A 5 or 6 $2 $2 ______

Die B 1, 2, 3 or 4 $2 $2 ______

Die B 5 or 6 $4 $0 ______

**Brown University Department of CLPS Study Debriefing**

This study investigates two possible factors that help us decide whether somebody's behavior is fair: their intentions, or the consequences of their behavior. Research into moral psychology suggests that intentions are a critical determinant of moral judgments. Economic research shows that people care about the intentions when judging whether an outcome is fair or not. However, outcomes also seem to matter a lot. This study is designed to test the relative importance of each factor, and how we make decisions when intentions and consequences conflict. For this, we look at fairness considerations in a situation where consequences are only partly linked to intentions (i.e. a participant chooses an outcome that will probably, but not definitely, occur).

How was this tested?

In this study, participants were divided into 2 groups. Some people played in the roll of “roller”, and other people played in the role of “responder”. The roller was asked to split an amount of money between herself and the responder. If you were the responder, you were asked how you would reward or punish the roller for how she allocated the money between the two of you. Critically, the roller could *attempt* to keep all the money to themselves or to split it between themselves and the responder, but this attempt failed 1/3 of the time according to the roll of a die.

Hypothesis and main questions:

Our experiment is designed to test whether people punish and reward according to what the roller *attempts*, or instead according to what *actually happens*. We hypothesize that people will reward and punish mostly on the basis of what actually happens.

Why is this important to study?

Moral judgments play a critical role in our daily lives. But what do we actually give more importance to, the intentions of an act or the consequences of it? A growing body of research suggests that many of the moral judgments that we make operate intuitively—without conscious access or understanding of the underlying principles. Understanding these intuitive moral principles gives us a valuable window into our own psychology, and also can help us make more informed moral choices. Similarly, understanding how third parties weigh intentions and outcomes has impact on what factors mediators and jurors in the court system consider the most when making their decisions.

What if I want to know more?

If you are interested in learning more about our moral psychology, you wish to consult:

Cushman, F.C. (2006). The role of reasoning and intuition in moral judgments: Testing three principles of harm. Psychological Science, 17 (12).

Cushman, F. A., Dreber, A., Wang, Y., & Costa, J. (2009). Accidental outcomes guide punishment in a 'trembling hand' game. PLoS One 4 (8): e6699.doi:10.1371/journal.pone.0006699.

If you would like to receive a report of this research when it is completed (or a summary of the findings), please contact Fiery Cushman (Fiery_Cushman@brown.edu).

If you have concerns about your rights as a participant in this experiment, please contact Susan Carton-Lopez (401) 863-9206 (Susan_Carton-Lopez@brown.edu), Assistant Director of the Research Protections Office.
