# Supplementary material for: To Punish or to Leave: Distinct Cognitive Processes Underlie Partner Control and Partner Choice Behaviors
Source: PLoS One. 2015 Apr 27;10(4):e0125193. doi: 10.1371/journal.pone.0125193 (PMC4411127; doi:10.1371/journal.pone.0125193)
Supplement: S2 File — Materials used in the Partner Choice condition. (DOC) [file pone.0125193.s002.doc]

**Experiment Script**

Subjects are welcomed and guided to opposite sides of the room in alternating order.

Experiment welcomes subjects, says:

- You will be handed instructions, they are complicated, please read them carefully.
- Experimenter will go over things verbally, please wait until after that to ask questions.
- Please try to keep a “poker face” during this experiment, the only interactions between participants should be on paper handed back and forth by experimenters.

All subjects receive instructions, read them.

Experimenter reviews instructions, saying:

- In this game you will interact with one other person in this room now, but that interaction will be completely anonymous.
- People are paired by roller/responder – identify which side of the room is which.
- Roller must allocate $4 between self and responder
- Choice of two die, explain how the die work
- Responder can either choose to stay with the same roller for the next round, or switch to a different roller
- *Totally up to responder to decide. Decide for yourself.*
- Responder must make decision for all possible outcomes in advance.
- We collect forms, work out what happens, and report back to both players
- Second round commences, same as first round

All subjects receive response sheet for round 1 and fill it out.

Experimenters collect sheets, compute outcomes, and return feedback sheets.

All subjects receive response sheet for round 2 and fill it out.

Experimenters collect sheets, compute outcomes, and return feedback sheets.

Everyone is paid in an envelope and leaves.

Thank you for participating in this experiment.

Please read the following instructions very carefully. If anything is unclear, please ask the experimenter for clarification.

In this experiment, pairs of people interact in a simple game up to two times. During each of these two “rounds”, one subject divides $4 between themselves and the other subject. This person is called the “roller” (you’ll see why, soon). The other subject then responds to that division. This person is called the “responder”.

Today, you are going to play the role of a “responder”. On the first round of this experiment we will randomly pair you with one of the rollers who is in the room right now. However, you do not know which person is your roller, and the roller does not know which responder you are. We will keep your identity, and the roller’s identity, completely anonymous.

At the beginning of each game, there is $4 to be divided between you and the Roller. The Roller will determine how to make this division. In order to do so, the roller can choose which of two die to roll: A or B. The roller can choose either one. Here’s how the die work:

Die A: If the roller rolls a 1, 2, 3 or 4, then the Roller gets all $4.

If the roller rolls a 5 or 6 and, then the Roller gets $2 and you get $2.

Die B: If the roller rolls a 1, 2, 3 or 4, then the Roller gets $2 and you get $2.

If the roller rolls a 5 or 6, then the Roller gets all $4.

After the die has been rolled, you will then have the chance to decide whether you want to stick with the same roller on the next round, or switch and be randomly assigned to a new roller on the next round. It is up to you whether you want to stick or switch, and if it is possible to grant your request, the experiment will do so. The roller will be informed of your decision to stick or switch.

Please turn the page.

Round 1 - Responder Subject ___

**For you to fill out:**

You can choose whether you want to keep playing with the same Roller on the next round, or switch to a new Roller. Indicate your choice given each possible outcome of this round.

**Roller The die Roller**

**rolls: comes up: gets: You get: Next game:**

Die A 1, 2, 3 or 4 $4 $0 Same Roller / New Roller

Die A 5 or 6 $2 $2 Same Roller / New Roller

Die B 1, 2, 3 or 4 $2 $2 Same Roller / New Roller

Die B 5 or 6 $4 $0 Same Roller / New Roller

**For the experimenter to fill out:**

In Round 1, the Roller chose die: ______

The die came up: ______

Your attempted response was: Same Roller / New Roller

This response did / did not occur.

Round 2 - Responder Subject ___

**For you to fill out:**

You can choose whether you want to keep playing with the same Roller on the next round, or switch to a new Roller. Indicate your choice given each possible outcome of this round.

**Roller The die Roller**

**rolls: comes up: gets: You get: Next game:**

Die A 1, 2, 3 or 4 $4 $0 Same Roller / New Roller

Die A 5 or 6 $2 $2 Same Roller / New Roller

Die B 1, 2, 3 or 4 $2 $2 Same Roller / New Roller

Die B 5 or 6 $4 $0 Same Roller / New Roller

**For the experimenter to fill out:**

In Round 2, the Roller chose die: ______

The die came up: ______

Your attempted response was: Same Roller / New Roller

This response did / did not occur.

Some Final Questions

1. Which die would you choose if you were the Roller? Why would you make that choice?

2. Which die did the Roller choose? Why do you think they made that choice?

3. What were the factors you considered when deciding whether or not to play the same Roller again?

4. We would like to know whether you found any of the decisions about staying or switching to be particularly difficult. Please put a check mark by any decision(s) you found particularly difficult:

Roller rolls: The die comes up: Roller gets: You get: Difficulty:

Die A 1, 2, 3 or 4 $4 $0 ______

Die A 5 or 6 $2 $2 ______

Die B 1, 2, 3 or 4 $2 $2 ______

Die B 5 or 6 $4 $0 ______

5. What is your gender? Please circle one: Female Male

6. What is your age in years? _____

7. What is your occupation? If you are a student, what is your primary field of study?

Thank you for participating in this experiment.

Please read the following instructions very carefully. If anything is unclear, please ask the experimenter for clarification.

In this experiment, pairs of people interact in a simple game two times. During each of these two “rounds”, one subject divides $4 between themselves and the other subject. This person is called the “roller” (you’ll see why, soon). The other subject then responds to that division. This person is called the “responder”.

Today, you are going to play the role of a “roller”. On the first round of this experiment we will randomly pair you with one of the responders who is in the room right now. However, you do not know which person is your responder, and the responder does not know which roller you are. We will keep your identity, and the responder’s identity, completely anonymous.

At the beginning of each game, there is $4 to be divided between you and the responder. You will determine how to make this division. In order to do so, you can choose which of two die to roll: A or B. You can choose either one. Here’s how the die work:

Die A: If you roll a 1, 2, 3 or 4, then you get all $4.

If you roll a 5 or 6, then you get $2 and the responder gets $2.

Die B: If you roll a 1, 2, 3 or 4, then you get $2 and the responder gets $2.

If you roll a 5 or 6, then you get all $4.

After the die has been rolled, the responder will then have the chance to decide whether they want to stick with the same roller (you) on the next round, or switch and be randomly assigned to a new roller (not you) on the next round. It is up to the responder whether they want to stick or switch, and if it is possible to grant their request, the experiment will do so. You will be informed of their decision to stick or switch.

Please turn the page.

Round 1 - Roller Subject ___

**For you to fill out:**

Please indicate which die you would like to roll. Circle one: **Die A Die B**

After you roll the die, please write down the number that came up here: ______

**For the experimenter to fill out:**

In Round 1, you chose die: ______

The die came up: ______

The responder’s attempted response was: Same Roller (you) / New Roller (not you)

This response did / did not occur.

Round 2 - Roller Subject ___

**For you to fill out:**

Please indicate which die you would like to roll. Circle one: **Die A Die B**

After you roll the die, please write down the number that came up here: ______

**For the experimenter to fill out:**

In Round 2, you chose die: ______

The die came up: ______

The responder’s attempted response was: : Same Roller (you) / New Roller (not you)

This response did / did not occur.

Subject ___

Some Final Questions

1. Which die did you choose? Why would you make this choice?

2. How did the other player respond to your choices? What factors do you think the other player was considering, and how did they make their decisions?

3. What is your gender? Please circle one: Female Male

4. What is your age in years? _____

5. What is your occupation? If you are a student, what is your primary field of study?

Roller as Responder Subject ___

**For you to fill out:**

You can choose whether you want to keep playing with the same Roller on the next round, or switch to a new Roller. Indicate your choice given each possible outcome of this round.

**Roller The die Roller**

**rolls: comes up: gets: You get: Next game:**

Die A 1, 2, 3 or 4 $4 $0 Same Roller / New Roller

Die A 5 or 6 $2 $2 Same Roller / New Roller

Die B 1, 2, 3 or 4 $2 $2 Same Roller / New Roller

Die B 5 or 6 $4 $0 Same Roller / New Roller

Some Final Questions

1. What were the factors you considered when deciding whether or not to play the same Roller again?

2. We would like to know whether you found any of the decisions about staying or switching to be particularly difficult. Please put a check mark by any decision(s) you found particularly difficult:

Roller rolls: The die comes up: Roller gets: You get: Difficulty:

Die A 1, 2, 3 or 4 $4 $0 ______

Die A 5 or 6 $2 $2 ______

Die B 1, 2, 3 or 4 $2 $2 ______

Die B 5 or 6 $4 $0 ______

**Brown University Department of CLPS Study Debriefing**

This study investigates two possible factors that help us decide whether somebody's behavior is fair: their intentions, or the consequences of their behavior. Research into moral psychology suggests that intentions are a critical determinant of moral judgments. Economic research shows that people care about the intentions when judging whether an outcome is fair or not. However, outcomes also seem to matter a lot. This study is designed to test the relative importance of each factor, and how we make decisions when intentions and consequences conflict. For this, we look at fairness considerations in a situation where consequences are only partly linked to intentions (i.e. a participant chooses an outcome that will probably, but not definitely, occur).

How was this tested?

In this study, participants were divided into 2 groups. Some people played in the roll of “roller”, and other people played in the role of “responder”. The roller was asked to split an amount of money between herself and the responder. Then, the responder had the chance to either **stay** with the same roller, or **switch** to a new roller.

Hypothesis and main questions:

Our experiment is designed to test whether people decide to stay or swich according to what the roller *attempts*, or instead according to what *actually happens*. We hypothesize that people will stay or switch mostly on the basis of what the roller attempts to do.

Why is this important to study?

Moral judgments play a critical role in our daily lives. But what do we actually give more importance to, the intentions of an act or the consequences of it? A growing body of research suggests that many of the moral judgments that we make operate intuitively—without conscious access or understanding of the underlying principles. Understanding these intuitive moral principles gives us a valuable window into our own psychology, and also can help us make more informed moral choices. Similarly, understanding how third parties weigh intentions and outcomes has impact on what factors mediators and jurors in the court system consider the most when making their decisions.

What if I want to know more?

If you are interested in learning more about our moral psychology, you wish to consult:

Cushman, F.C. (2006). The role of reasoning and intuition in moral judgments: Testing three principles of harm. Psychological Science, 17 (12).

Cushman, F. A., Dreber, A., Wang, Y., & Costa, J. (2009). Accidental outcomes guide punishment in a 'trembling hand' game. PLoS One 4 (8): e6699.doi:10.1371/journal.pone.0006699.

If you would like to receive a report of this research when it is completed (or a summary of the findings), please contact Fiery Cushman (Fiery_Cushman@brown.edu).

If you have concerns about your rights as a participant in this experiment, please contact Susan Carton-Lopez (401) 863-9206 (Susan_Carton-Lopez@brown.edu), Assistant Director of the Research Protections Office.
